# Light People: Professor Cheng Zhang

**DOI:** 10.1038/s41377-023-01291-3

**Published:** 2023-10-03

**Authors:** Siqiu Guo

**Affiliations:** Light Publishing Group, Changchun Institute of Optics, Fine, Mechanics and Physics, Chinese Academy of Sciences, 3888 Dong Nan Hu, Road, Changchun, 130033 China

## Abstract

Nanophotonics has emerged as a cutting-edge interdisciplinary research field today. Its primary objective is to leverage the interaction between light and matter at the wavelength and sub-wavelength scales, with the purpose of designing and manufacturing miniaturized, multifunctional, and high-performance optical devices and systems. Professor Cheng Zhang from Huazhong University of Science and Technology has dedicated his career to nanophotonic device research. His work encompasses a wide range of areas, including plasmonic devices, optical metamaterials, and metasurfaces. Through the design of innovative artificial electromagnetic structures and the exploration of emerging nanofabrication techniques, Professor Cheng Zhang has effectively achieved versatile control over various properties of electromagnetic waves, including amplitude, phase, and polarization states. Furthermore, his research extends to the continuous exploration of novel optical phenomena, aimed at realizing high-performance engineering applications. In this edition of Light People, we will take you deep into the world of Professor Cheng Zhang, a young scientist exemplifying the spirit of innovation, relentless improvement, and unwavering pursuit of excellence. You will discover how he has overcome numerous challenges in the realm of nanophotonic research.

**Short Bio:** Dr. Cheng Zhang is a professor at School of Optical and Electronic Information, Huazhong University of Science and Technology (HUST), Wuhan, China. He obtained his B.S. degree in Electrical Science and Technology from Shandong University in 2010, and Ph.D degree in Electrical Engineering from the University of Michigan-Ann Arbor in 2016. From 2016 to 2020, he was a post-doc researcher at National Institute of Standards and Technology (NIST), Gaithersburg, MD, USA. He now leads an interdisciplinary research team working on cutting-edge projects aimed at the exploitation of nanophotonic materials, devices and fabrication techniques for novel information, sensing and energy harvesting applications.

**Figure:**
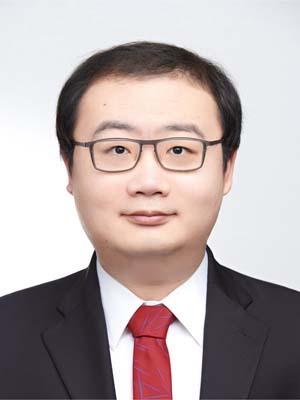

**1. The first time we met was at the unveiling ceremony of the Light-Wuhan office. I am very happy to hear your wonderful talk during the meeting. Our first impression of you was young and talented, so what was your first impression of** ***Light: Science & Applications*** **(*Light*)?**

Thank you and I was delighted to meet you and your colleagues in Wuhan this March too. I became acquainted with *Light* around 2013 when I was in the third year of my Ph.D. at the University of Michigan. I noticed that several important works related to my research appeared in the journal, and one of my friends also had his work published in *Light*. Based on my readings and my friend’s feedback, I perceived *Light* as a highly selective journal that seeks truly novel and important findings. I hoped that one day that I could have the opportunity to publish my work in *Light* as well.

During our meeting in Wuhan, I was impressed by your diligent work attitude. I observed you and the other editors actively responding to emails and managing submissions even during the meeting breaks. Your enthusiasm for staying updated on the latest developments in various fields of optics and attracting high-quality submissions for the journal was evident. I believe this dedication is one of the factors that contributed to *Light* quickly establishing itself as a leading journal after launch.

I would also like to say that *Light* has evolved beyond being solely an academic journal for researchers in optics. It has transformed into a vibrant community that facilitates the connections and sharing of research outcomes among optics researchers. Light Publishing Group has successfully launched two other impactful journals: *eLight* and *Light: Advanced Manufacturing*. Additionally, the establishment of 19 offices worldwide demonstrates the global reach and influence of *Light*. Moreover, *Light* organizes annual *Light* conferences and other special academic events regularly, providing valuable opportunities for networking and knowledge exchange. *Light* WeChat official account, “Chinese Optics”, has become one of my primary sources for staying updated on the latest advancements in the field. The articles posted on your account showcase new works published by *Light* and other journals, making it an invaluable channel for me to learn about the field’s latest developments.

**Figure:**
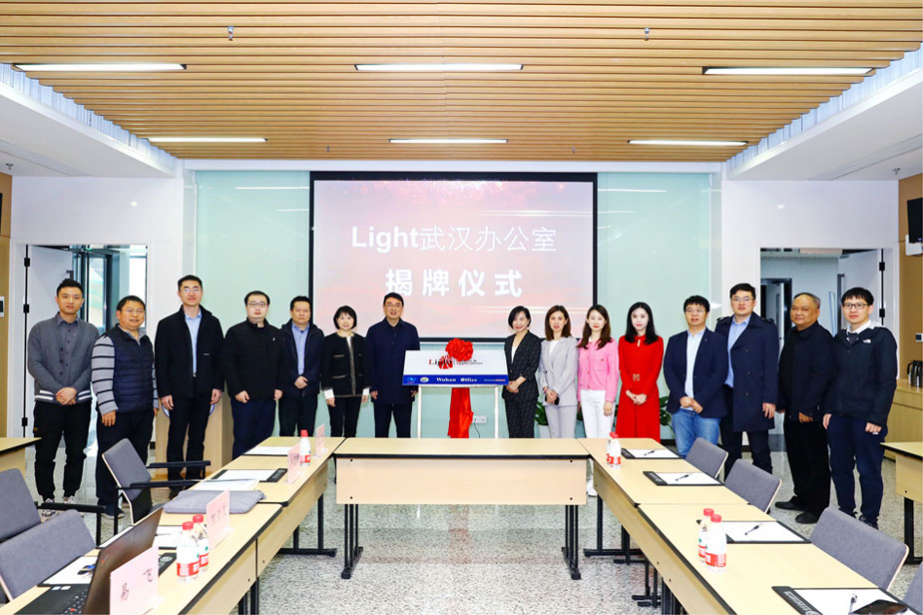
The unveiling ceremony of Light-Wuhan Office

**2. Could you introduce your research on optical meta-devices during your Ph.D and post-doc period?**

During my Ph.D study at Umich, I focused on the field of metamaterials and metasurfaces, which remains the primary research focus of my group to this day. Throughout my doctoral research, I and my collaborators worked on several plasmonic metamaterials and metasurfaces, which are devices comprised of sub-wavelength metallic nano-antennas. We developed one-dimensional (1D) silver (Ag) groove-based structural color generating metasurfaces capable of producing angle-insensitive reflection colors^1^. Additionally, we explored cascaded bianisotropic gold (Au) metasurfaces, which allowed for novel control over the state of polarization and propagation direction of incident light^2–4^.

During my post-doctoral training at NIST, my research focus remains centered on metamaterials and metasurfaces, with a particular emphasis on those composed of low-loss dielectric nanostructures. One important work is realizing the first metasurface optics operating down to the deep ultraviolet (deep-UV) region^5^, which I will elaborate on in the following text. Another milestone work is achieving light field shaping in the temporal domain based on dielectric metasurfaces^6^.

While the exploration of optical metasurfaces gained momentum around 2010, much of the research initially concentrated on shaping light fields across the spatial domain. These investigations yielded an array of high-performance metasurfaces capable of various light manipulation tasks, including focusing, steering, filtering, and diffracting. However, for a complete description of an optical field, we need to consider both its spatial and temporal characteristics. My collaborators and I showed, for the first time, that the metasurface technology could be employed to sculpt optical fields temporally. As experimental demonstrations, we designed and fabricated metasurfaces consisting of silicon (Si) nanopillars, and employed the samples in a Fourier-transform setup to shape the temporal profile of a near-infrared femtosecond pulse. Leveraging the inherent flexibility of metasurface design, we were able to manipulate, simultaneously and independently, the amplitude and phase of the constituent frequency components of the femtosecond pulse, and realized an array of finely tailored pulse-shaping operations, including splitting, compression, chirping, and higher-order distortion. This study not only showcases a viable platform for miniaturized optical technology with advanced time-dependent functionality but also proves the immense potential of metasurface technology in orchestrating multi-dimensional manipulation of optical fields in a spatiotemporal manner.

**3. I notice that one important part of you Ph.D research is on ultra-thin doped Ag films. How did you initiate this study?**

The study on “ultra-thin doped Ag” is an important part of my Ph.D research, but it is indeed an intriguing and unexpected part. When I and my lab mates were working on the Ag-based structural color devices^1^, we noticed that the devices exhibited subpar performance in the blue region, likely due to Ag’s higher loss in that wavelength range. Recognizing that aluminum (Al) is a superior plasmonic material for the blue region, we conceived the idea of combining Ag with Al to obtain improved responses in the blue region while maintaining similar responses in the green and red regions. We thought this idea is so cool as it allowed us to get the best of both worlds! However, despite numerous trials in the cleanroom, the results were not as promising as we had hoped, as many research endeavors often encounter.

Nevertheless, during the process of mixing Ag with Al, I extensively surveyed different literatures on the growth and characterization of metallic films. I learnt that Ag struggles to form stable and ultra-thin (<15 nm) films due to the intrinsic 3D growth mode of Ag atoms. Even though doping Ag with Al did not yield the desired outcome for our initial objective, I became curious about whether such doping could facilitate the formation of ultra-thin Ag films.

I distinctly recall performing the first experiment in the lab after midnight, and to my surprise, it was a resounding success on the first attempt. Subsequently, the process became much more straightforward. We achieved low-loss, ultra-smooth and ultra-thin (down to 6 nm) continuous films with significantly enhanced long-term and thermal stability^7,8^. We further explored different doping options and found that various doping materials aided in ultra-thin Ag film formation^9^. We further incorporated these doped Ag films in a wide range of optoelectronic devices, including hyperbolic metamaterials^10^, plasmonic waveguides^11^, transparent conductors^12,13^, organic solar cells^14^, light-emitting diodes^9^, and more.

**4. At the beginning of 2020, you returned to your homeland with a dream and formed your own research team. Please introduce your research direction and the latest research progress**.

My research team currently comprises over 20 members, including post-doctoral researchers, Ph.D and master students, as well as undergraduate students. Our primary focus lies in investigating various types of light-matter interactions at the subwavelength scale, alongside studying the associated devices, systems, and applications.

One major area of interest for us is metasurface-empowered light field modulation. Metasurfaces are planar arrays of subwavelength electromagnetic nano-antennas that mimic the functionality of traditional refractive, reflective, or diffractive optical components. They achieve this by individually tailoring the amplitude, phase, or polarization transformations of incident light. Metasurfaces hold tremendous potential for building the next-generation optical systems with reduced size and enhanced functionality. Our research seeks to explore new applicable wavelength regions for metasurface technology, such as the UV and deep-UV spectra. Additionally, we investigate related device designs, fabrication techniques, and practical applications.

We are delving into novel mechanisms for utilizing metasurfaces to achieve multi-dimensional light field control. This entails independently and simultaneously manipulating multiple parameters of the light field, including amplitude, phase, polarization, and more. We leverage these developed metasurfaces for advanced imaging and display applications. For instance, we are working on new augmented reality/virtual reality display systems that offer reduced footprint and enhanced functionality^15,16^. Additionally, we explore photonic chips capable of optical computing and image processing, as well as structural colors exhibiting unique hues and saturations^17^. I look forward to sharing our new research results in *Light* in the near future.

**Figure:**
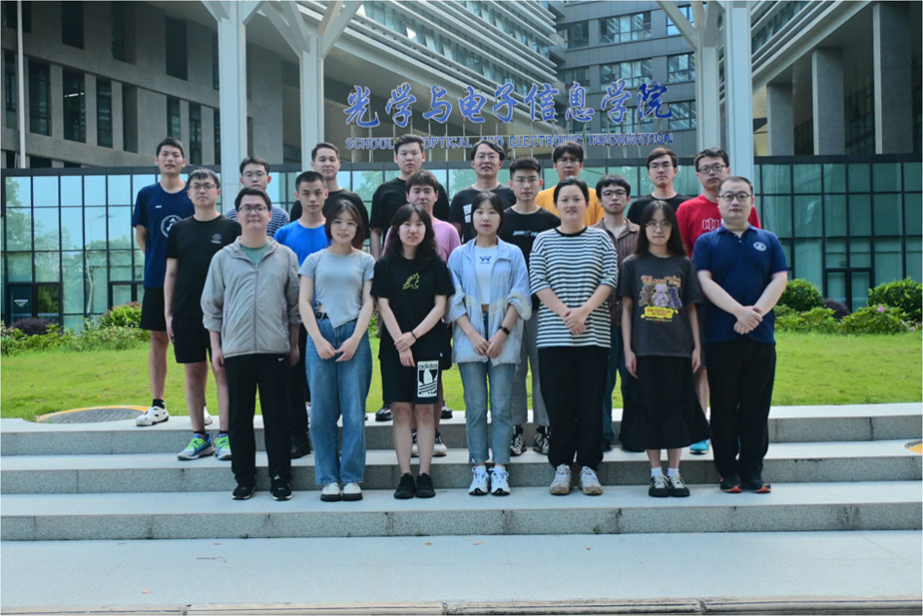
Group photo of team members

**Figure:**
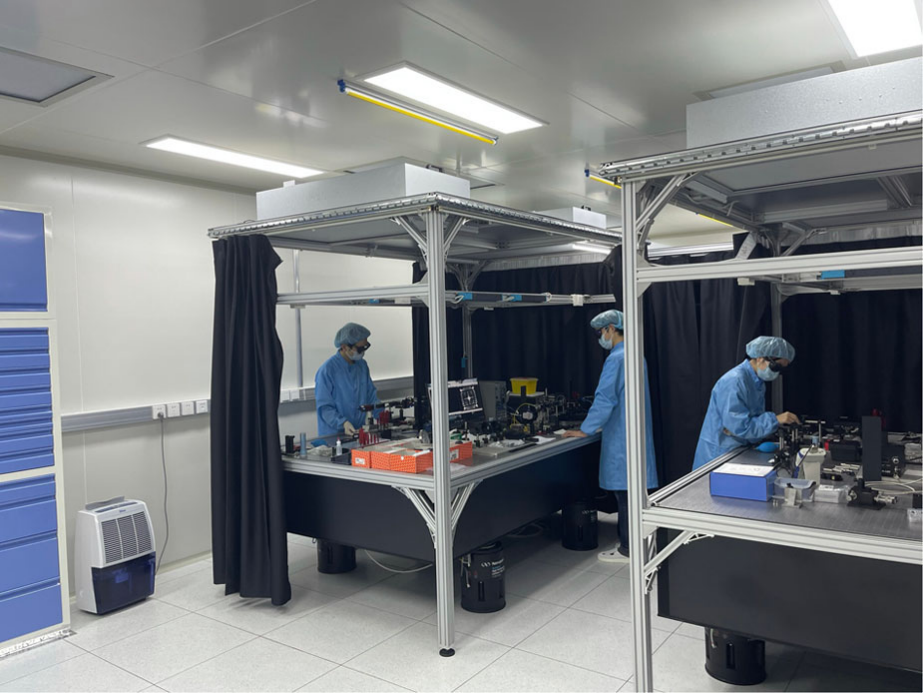
Inside look of the optical characterization lab

**5. It was an honor to visit your laboratory last time we met. Many of your important scientific research ideas were implemented here. Can you briefly introduce the laboratory you built yourself?**

Our lab specializes in studying various micro- and nano-scale optical devices, covering a broad wavelength range from UV to IR. To facilitate our research, we have access to a range of light sources, including super-continuum lasers, gas lasers, and solid-state lasers. Additionally, we possess an array of characterization tools such as microscopes, angle-resolved reflection/transmission spectrum measurement setups, spectrometers, power meters, beam profilers, polarimeters, scientific cameras, and more. We have also installed several workstations, so that the group members can easily perform various kinds of electromagnetic (EM) computation using either commercial EM softwares or their own codes.

Furthermore, we have designed and constructed several customized in-house characterization systems. These systems include holographic imaging systems, which enable us to capture and analyze holographic images. We also have built metalens characterization and imaging systems that facilitate the assessment of metalens performance and imaging capability. Additionally, we have beam deflection measurement systems, allowing us to measure the deflection angles of light beams. Lastly, we have Fourier space imaging systems that enable us to study the spatial frequency contents of optical signals.

**Figure:**
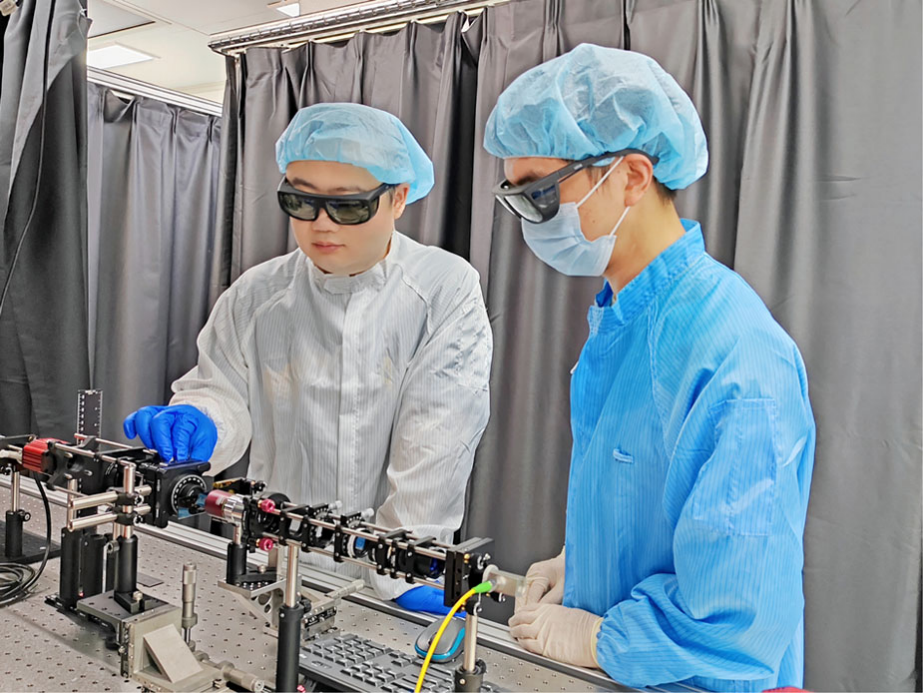
Prof. Cheng Zhang and Mr. Zhelin Lin (Ph.D student) work on metalens characterization

**6. How do you admit graduate students? Are there any specific criteria?**

Usually, around summer time of year, I receive many inquiries from students seeking graduate study opportunities in my research group. I will choose to engage in discussions with some of the candidates. Apart from briefly discussing their previous coursework and extracurricular experiences, I usually ask the following questions in greater detail:

1. How did you learn about our lab, and what specific aspects of our group sparked your interest in joining?
2. What are your career goals, and what type of job do you envision pursuing after graduation?
3. Do you have any questions for me? Feel free to ask any type of question.

It’s interesting to note that there are no fixed answers to these questions, and I always hear various interesting responses. I recall a student who mentioned that while browsing our group website, he came across photos of our birthday parties and get-together dinners. The cozy and happy atmosphere depicted in those photos convinced him that our group was where he wanted to work. Many students ask various types of interesting questions, ranging from “how does a typical day in the lab look like”, “how is the job market after graduation” to “what is the next big thing in nanophotonics”. There were also students who have responded that they did not have any questions to ask, and I must admit, I felt a bit disappointed with that answer.

When asking these questions, which may not directly relate to their research and coursework, I’m not seeking specific answers. Instead, I aim to understand how candidates gather and process information, as well as their ability to ask thought-provoking questions based on the information they have collected. These abilities are crucial in our daily research endeavors. Additionally, through these discussions, I assess their capacity for effective life planning and evaluate whether they possess qualities and characteristics that align with our research group’s objectives.

**Figure:**
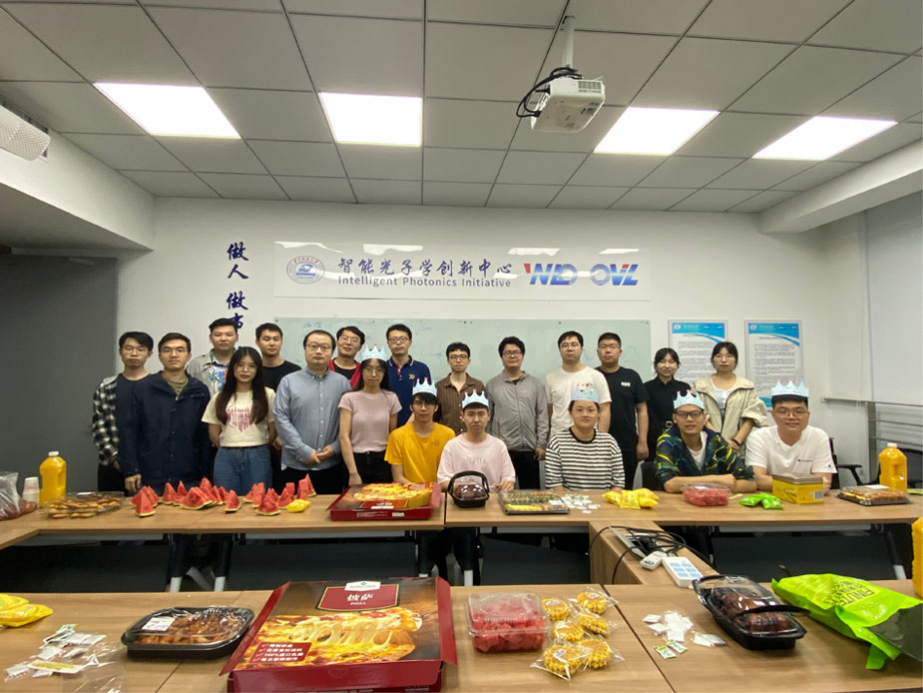
Quarterly group birthday party

**7. As a graduate advisor, you have supervised many graduate students. What abilities do you focus more in cultivating students?**

In my opinion, the minimum requirement for qualified graduate students is a thorough understanding of the foundational knowledge within their field, coupled with a proficiency in basic simulation or experimental skills. This combination equips them to confidently embark on research projects and initiate their own scientific explorations. The student is better to have a strong inclination towards self-directed learning and a persistent motivation to stay informed about new developments in the field. Such commitment ensures a continuous expansion of their knowledge base and refinement of their research skills. Besides of these basic expectations, there are abilities to which I will pay special attention in working with my graduate students.

1. **Critical Thinking**: The ability to analyze and dissect complex problems, evaluate various perspectives, and formulate innovative solutions is indispensable for achieving success in research. As Mencius wisely advises, “Do not blindly accept everything written in books.” While referencing textbooks and literature can be beneficial, my ambition is for students to consider all pertinent elements within their practical contexts and unearth answers stemming from their own wisdom and discernment. This approach paves the way for genuine scientific breakthroughs, truly original in nature.
2. **Adaptability & Flexibility**: The scientific landscape is in a constant state of flux, rendering it nearly impossible to foresee and plan for every aspect when commencing a new research project. It is commonplace to encounter some unforeseen circumstances during an experiment. Consequently, individuals must possess a readiness to embrace novel methodologies and approaches in response to the unfolding developments and challenges that arise throughout the course of the research endeavor.
3. **Effective Communication**: Clear and efficient communication skills, both in writing and verbally, is vital for conveying research findings and collaborating with fellow researchers. Furthermore, I feel that, particularly for graduate students, the cultivation of effective communication extends beyond crafting well-structured papers or delivering engaging presentations.
  In particular, I hold the expectation that students should adeptly communicate with their advisors and co-workers the status of their research projects, their needs, requirements, goals, etc. A notable instance is when a research project encounters a bottleneck, the timeliness of resolving the issue often largely relies on the student’s capacity to logically and comprehensibly describe the prevailing situation and the trials undertaken. I also encourage students to openly communicate their needs to me, including but not limited to equipment requisitions for their projects, the intention to attend conferences or other academic events, the interest to explore some new ideas, the desire to establish connections with individuals I may be acquainted with, etc. In my role as their mentor and probably their closest collaborator over several years, I recognize my duty to offer such multifaceted support.
4. **Effective Management**: In this context, management doesn’t exclusively mean leading a team but rather encompasses the skill of self-management. This includes proficient time management, good motion management, adept relationship management, and efficient process management, etc.

Efficient time management is crucial for optimizing productivity and executing research tasks with precision. It involves setting priorities, allocating resources efficiently, and adhering to well-defined schedules. The individual should also try to keep a good balance between work and relaxation. In our daily lives, we frequently encounter difficulties and unexpected situations, trivial or significant. The practice of effective motion management equips us to sustain a constructive working state even amidst these challenges, thereby safeguarding both our professional efficiency and personal well-being. Relationship management involves establishing and maintaining meaningful connections with peers, mentors, collaborators, etc. The ability to communicate and negotiate effectively, collaborate harmoniously, and navigate interpersonal dynamics plays an essential role in both personal growth and research success over a long run. Process management entails the strategic coordination and optimization of workflows, methodologies, and procedures, involving aspects such as identifying bottlenecks, streamlining workflows, seeking resources, delegating tasks, and employing innovative strategies to enhance efficiency and achieve research milestones.

Indeed, these skills extend far beyond the realm of scientific research itself, but rather hold universal value across various professions. My aspiration is for students to cultivate and apply these skills during their time as graduate students in my group. Then, they are equipped with essential competencies that will serve as the cornerstone of their future professional careers.

**8. The paper you published in** ***Light*, “Low loss metasurface options down to the deep ultraviolet region”, has been cited for over hundred times**^5^**. Could you please introduce the innovation of this paper?**

This work is a milestone publication in my research career, and it fills me with immense honor to have the work published in *Light*. Within this study, we demonstrate metasurface optics operating down to the deep-UV region, a record-short wavelength region at that time, all while maintaining exceptional device performance.

I had the idea of trying UV metasurfaces shortly after my arrival at NIST in October 2016. Around that time, several dielectric materials had been demonstrated for constructing high-efficiency metasurfaces, including TiO_2_, Si, GaN. However, none of these materials were suitable for the UV because they have relative narrow bandgap values and exhibit strong absorption of UV light.

My hope was to discover a suitable material that could serve as a new platform for UV meta-optics. Moreover, the material is better to be applicable for a continuous and broad UV range into the deep UV. Large bandgap value is one factor to consider. In addition, the material needs to exhibit a high refractive index, preferably exceeding 2. A high refractive index facilitates the design of various metasurfaces based on different operating principles. Another vital consideration is the material’s amenability to nanofabrication, including the easy accessibility to the material and the feasibility of patterning it into subwavelength nanostructures while maintaining robust profiles. A combination of these requirements eliminates numerous potential candidate materials from consideration.

After many rounds of trial, we ultimately chose to use Hafnium Oxide (HfO_2_) for the construction of UV and deep-UV metasurface optics. HfO_2_ is a wide-bandgap (E_g_ ~5.8 eV) material and has been commonly exploited as a high-static-dielectric-constant (high-κ) material in integrated circuit fabrication. Nonetheless, its applications in photonics had largely been limited to optical coatings based on planar thin films at that time, in particular, due to the difficulty of patterning the material into high-aspect-ratio nanostructures.

To pattern HfO_2_ into high-aspect-ratio subwavelength structures, we developed a resist-based Damascene process. The designed metasurface pattern was first created in a resist layer through lithography, and then the open spaces in the resist template were filled with HfO_2_. A low-temperature thermal atomic layer deposition (ALD) recipe was developed to deposit high-optical-quality HfO_2_ films. Through back-etching and resist removal, high-aspect-ratio HfO_2_ nanopillars with good side-wall profiles were successfully obtained. Using the developed platform, we demonstrated an array of high-performance metasurface devices operating down to the deep-UV, including metalenses, metaholograms, and accelerating beam generators.

Finally, I am glad to say that another paper has just been accepted by *Light*, showcasing a new metasurface platform for the UV and visible regions based on Tantalum Pentoxide (Ta_2_O_5_). With its wide bandgap of 4.0 eV, Ta_2_O_5_ enables low-loss metasurface operation across the whole visible and near-UV spectrum, and a part of the mid-UV region. Instead of relying on the ALD-based Damascene process as in the case of HfO_2_, Ta_2_O_5_ can be deposited into large-area and high-quality films through commonly-available physical vapor deposition (PVD) methods, and patterned into high-aspect-ratio nanostructures using common fluorine-based RIE processes, all in a CMOS-compatible manner. Based on this material platform, we successfully implement a series of high-efficiency UV and visible metasurfaces offering a set of representative light-field modulation functionalities, including high-numerical lensing, holographic projection and structural color generation, with peak operational efficiencies close to 80%. I would like to emphasize that the HfO_2_ and Ta_2_O_5_ platforms are inherently complementary to each other. Together, they offer a dynamic duo of material options that have the potential to revolutionize a wide array of applications, spanning fields such as lithography, imaging, spectroscopy, and quantum information processing.

**9. What is your view on the future of micro-nano photonic devices, and what do you think is the next breakthrough of this field?**

There are various exciting ongoing developments in the area of micro-nano photonic devices, and *Light* has been a leading journal in reporting these groundbreaking advancements. Researchers are exploring a diverse array of new physical principles for device design, encompassing concepts like bound states in the continuum, parity-time symmetry, and topological optimization. Furthermore, intelligent inverse designs, powered by techniques such as neural networks and transformer frameworks, are ushering in an era of high-performance devices that complement and enhance traditional design approaches. The fusion of micro-nano photonic devices with conventional optical elements represents another compelling avenue. This synergy maximizes the strengths of both domains, promising innovative outcomes.

Efforts in device fabrication are directed toward new techniques that offer larger size, reduced cost, faster throughput, and higher precision. Emerging materials such as phase change materials, Van der Waals materials, and transition metal dichalcogenides are being explored. The distinct optoelectronic properties of these materials, including tunable refractive indices and intrinsic anisotropic responses, are fostering new horizons for novel device functionalities.

These micro-nano photonic devices are driving a spectrum of exciting applications, including advanced imaging and displaying, augmented reality (AR) / virtual reality (VR) / extended reality (XR), optical computing, quantum measurement, and more. These applications further stimulate the refinement of micro-nano photonic devices, creating a cycle of innovation that propels both device evolution and application expansion.

**Figure:**
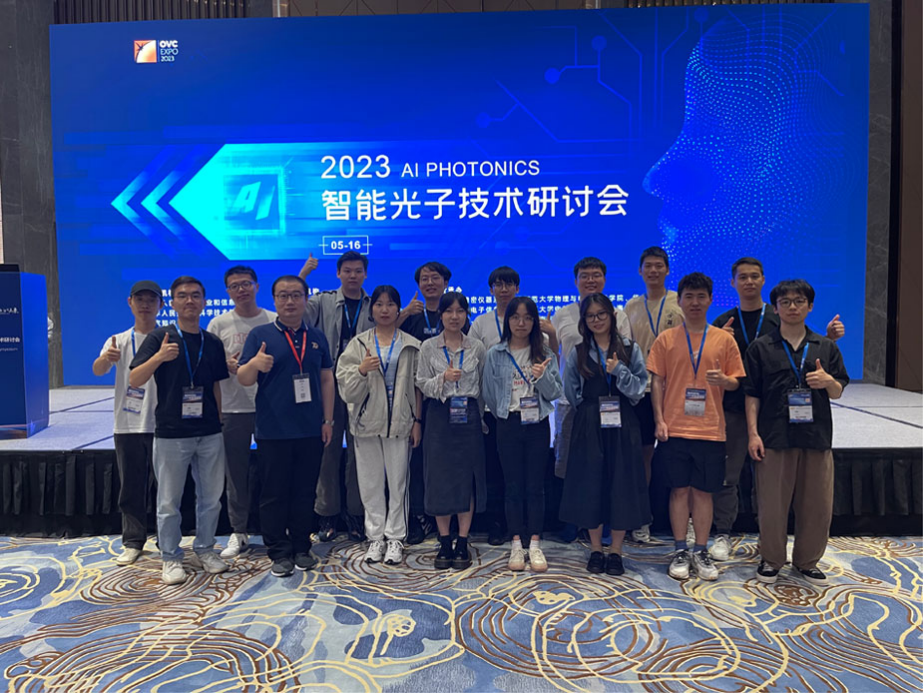
Group members attend the AI (artificial intelligence) Photonics Symposium in Optics Valley of China International Optoelectronic Exposition and Forum (OVC Expo 2023)

**10. Could you talk about your volunteer experience within the optics community**.

I have over 10 years of volunteer experience for the optics community since graduate school, taking on various roles such as OSA/SPIE student chapter president, OSA technical group chair, and conference organizer.

When I was a Ph.D student at Umich, I co-founded the OSA/SPIE joint student chapter, and served as its vice-president (2012–2014) and president (2014–2015). I worked closely with other chapter officers and hosted various types of events including new student orientation, faculty-student mixer, member bi-weekly meeting, Nano-Day outreach, etc. In addition, I served as the co-chair of the Engineering Graduate Symposium at University of Michigan in 2014, which is an annual campus wide conference where students from different engineering departments come together and present their research.

In 2016, I took on a new role within OSA and started to serve as the Event Leader of the OSA Nanophotonics Technical Group Executive Committee. My major responsibility is to organize networking and technical events for fellow OSA members. I successfully hosted three webinars on various topics including optical metamaterials and silicon photonics. In addition, I organized several special events at Conference on Lasers and Electro-Optics (CLEO) and Frontier in Optics (FiO) conferences. In particular, our technical group successfully introduced several new event formats, including 20 × 20 Talks and paired networking events, which have since been used throughout the OSA Technical Group program.

I was elected Group Chair in 2019, and had been serving this role till 2021. I continued the group’s momentum by leading the organization of poster sessions, technical webinars, panel discussions, and networking events. Our group regularly participated in the OSA Technical Group Poster Session at CLEO, which provides students and recent graduates with the opportunity to highlight their research. During the pandemic period, our group has organized several online events including academic webinars and panel discussions. Our group has also focused on providing opportunities for students and recent graduates in the field, by spending considerable time pairing senior members of the group with student mentees based on their research interests.

I have served as committee members or session organizers for over 20 optics conferences including Conference on Lasers and Electro-Optics (CLEO), Progress in Electromagnetics Research Symposium (PIERS), and International Photonics and OptoElectronics Meeting (POEM). I also participated in the organization of “Photonics Open Courses” and “Magic Photonics Online Forum”. I also help to review for over 40 journals including *Nature, Light: Science & Applications, eLight, Light: Advanced Manufacturing, Science Advances, Optica, Nano Letters*.

**Figure:**
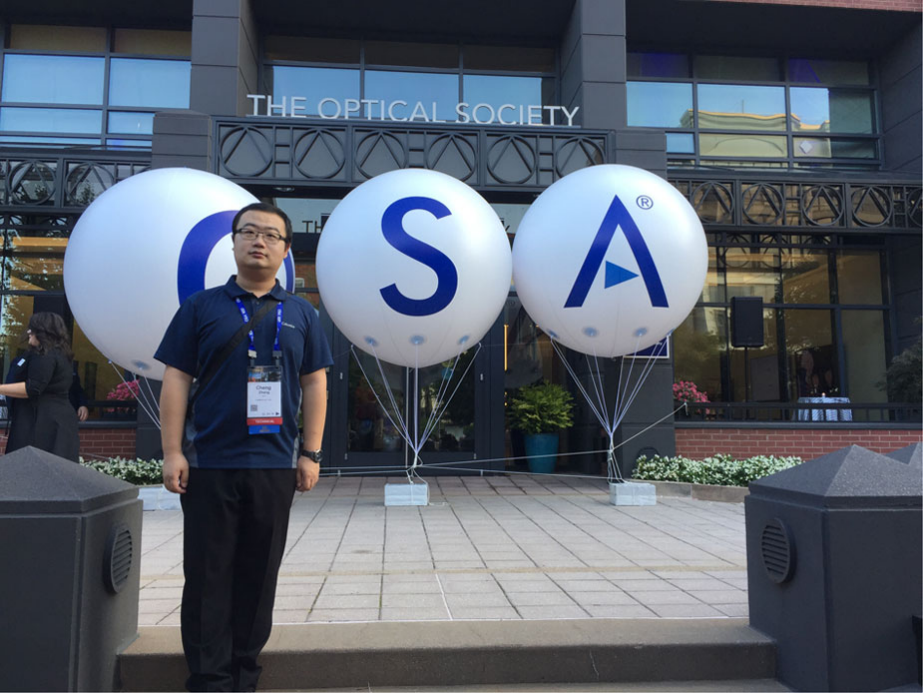
Photo taken at OSA headquarter in Washington DC (Sep. 2017)

**11. What obstacles or difficulties have you encountered in your research work? How did you overcome these difficulties?**

As an early-career principal investigator (PI), I encounter a wide range of difficulties and challenges in my daily research. These challenges can be trivial issues including unexpected tool malfunctions and forgetting to purchase necessary experiment parts ahead of time; or can be significant setbacks such as project progress delays and repeated rejections of proposals or papers. Moreover, budget constraints and the pressure to meet deadlines can add to the stress.

In my earlier years, especially during my school days, I would often blame myself for not performing or planning well when faced with such difficulties. It would leave me feeling nervous and down for days. In some cases, if I couldn’t find fault within myself, I would attribute the challenges to external factors or an unfavorable working environment. However, over time, and perhaps due to experiencing numerous failures (in jest), I have become more accepting of these challenges. I now understand that difficulties and challenges are an inherent part of our daily lives, including the scientific research journey. As long as we are actively pursuing our research goals, we are bound to encounter various obstacles. Some challenges can be anticipated, while others may seemingly arise out of nowhere. Furthermore, each of us has limitations in terms of time, energy, and abilities, so it is inevitable that there will be tasks we don’t execute well. We also cannot always choose our working conditions or the people we interact with, so facing non-ideal situations is a reality we have to accept.

However, acknowledging these realities does not mean that we are powerless. We can continually improve our skills and expand our knowledge, enabling us to handle difficulties more effectively. Additionally, as scientists and engineers, we have faith in the principles of science. If an experiment or plan fails, there must be underlying reasons for it. By employing logical thinking and analytical skills, we can investigate the cause and learn something new in the process. There are abundant resources available in books, literature, and the internet that can aid in the above problem-solving. This is the common advice I offer to my students when their projects encounter obstacles. Furthermore, maintaining professionalism and trustworthiness in our interactions with others helps to cultivate positive working relationships. While we can’t control the actions of others, we can strive to be reliable and supportive colleagues, always willing to lend a helping hand when needed.

Seeking help is also a valuable approach. I often reach out to senior or peer faculty members in my university or other institutes for advice on various matters, ranging from course teaching and experimental techniques to lab construction and funding applications. I am truly grateful for the generous assistance they provide. When I feel stressed or stuck in my work, I choose to spend time with family and friends, sharing a big meal, playing a game of badminton, or simply going for a walk after dinner, etc. These moments allow me to recharge and rejuvenate.

**12. What was the most exciting moment in your research career?**

When Imagination Turns into Reality, the most thrilling moments in my research journey are those when the imagination materializes into reality. These exhilarating moments occur when experiments, which have consumed considerable time and effort, finally yield substantive results or when audacious research concepts unexpectedly validate themselves. For the most part of a researcher’s daily life, it is likely to be characterized by a sense of routine and even moments of frustration, as you need to deal with many obstacles, big or tiny, foreseen or unexpected. However, the sheer delight of witnessing an experiment finally bear fruit not only justifies the challenges but also infuses me with renewed energy for the subsequent endeavors.

**13. Has anyone had a major influence on you in your career? In what way?**

I am deeply indebted to my mentors and collaborators who have indelibly shaped my career trajectory. I would like to take this opportunity to thank my Ph.D advisor, Prof. L. Jay Guo, and my post-doc advisor, Dr. Henri J. Lezec. They have not only provided a nurturing environment for me to explore novel ideas but also offered unwavering support when I faced challenges. They have behaved as role models for me. I have to admit that the manner in which I run my own research group and interact with my students is significantly influenced by them. I would also like to take this opportunity to thank my parents. Their unreserved encouragement and wholehearted support have been the wellspring of strength during moments of difficulty.

**Figure:**
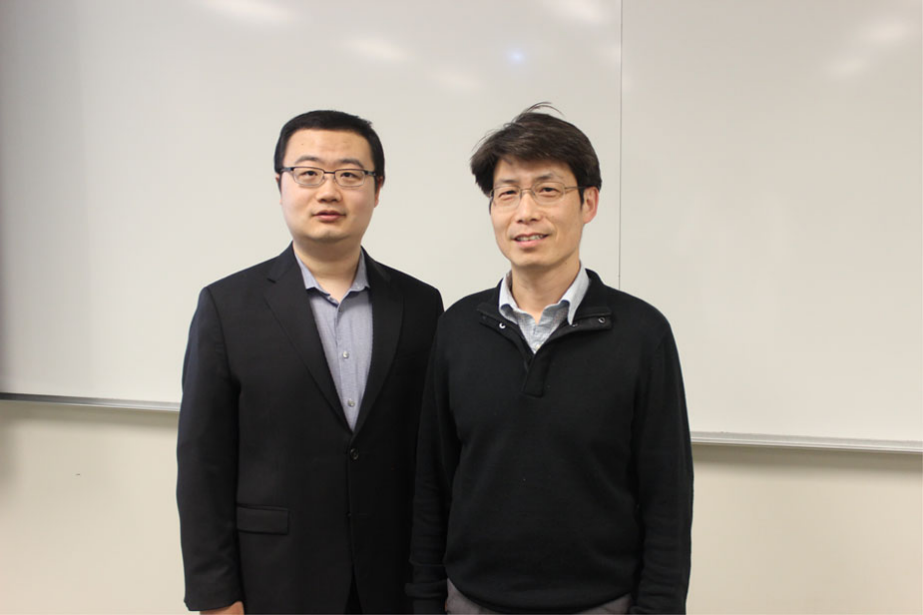
Photo with Ph.D advisor, Prof. L. Jay Guo (May 2016, at Ph.D defense)

**Figure:**
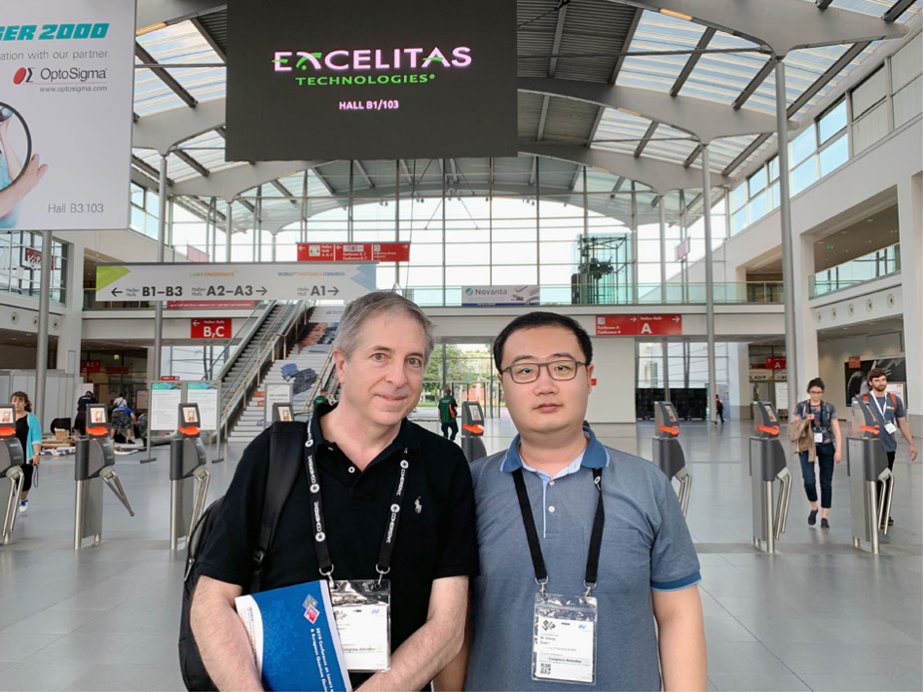
Photo with post-doc advisor, Dr. Henri J. Lezec (June 2019, at CLEO Europe, Munich)

**14. How do you balance your work and personal life?**

Well, honestly speaking, I am still learning to find the right balance between work and personal life. I feel that this is especially challenging for early-career researchers, as you need to take care of so many responsibilities, such as setting up labs, procuring equipment, securing funding, mentoring students, writing papers, etc. It can feel quite overwhelming at times.

I am trying to set certain “boundaries” between work and personal life, by designating specific hours for work-related tasks and reserving uninterrupted periods for personal activities and spending time with family and friends. Efficient time management and task delegation are also very helpful.

**15. What are your hobbies?**

Like many of my peers, I am fond of watching movies and television shows, listening to popular music, and reading novels. As my daily routine, I usually take a one-hour walk after dinner. Often, I take the walk inside our expansive university campus, which have many adorable animals. On occasion, I bring along some food for the cats and ducks. Feeding time becomes a very relaxing activity.

I like to visit museums, art exhibitions, and historic buildings. Immersing myself in these artworks brings about a unique sense of relaxation and serves as a wellspring of inspiration. When I visit a new place, I have the habit of bringing back a few magnets that showcase the distinctive landmarks of the place. These magnets serve as my personal keepsakes, preserving cherished memories of my travels. Over the years, my collection has grown to include more than 100 unique pieces.

**Figure:**
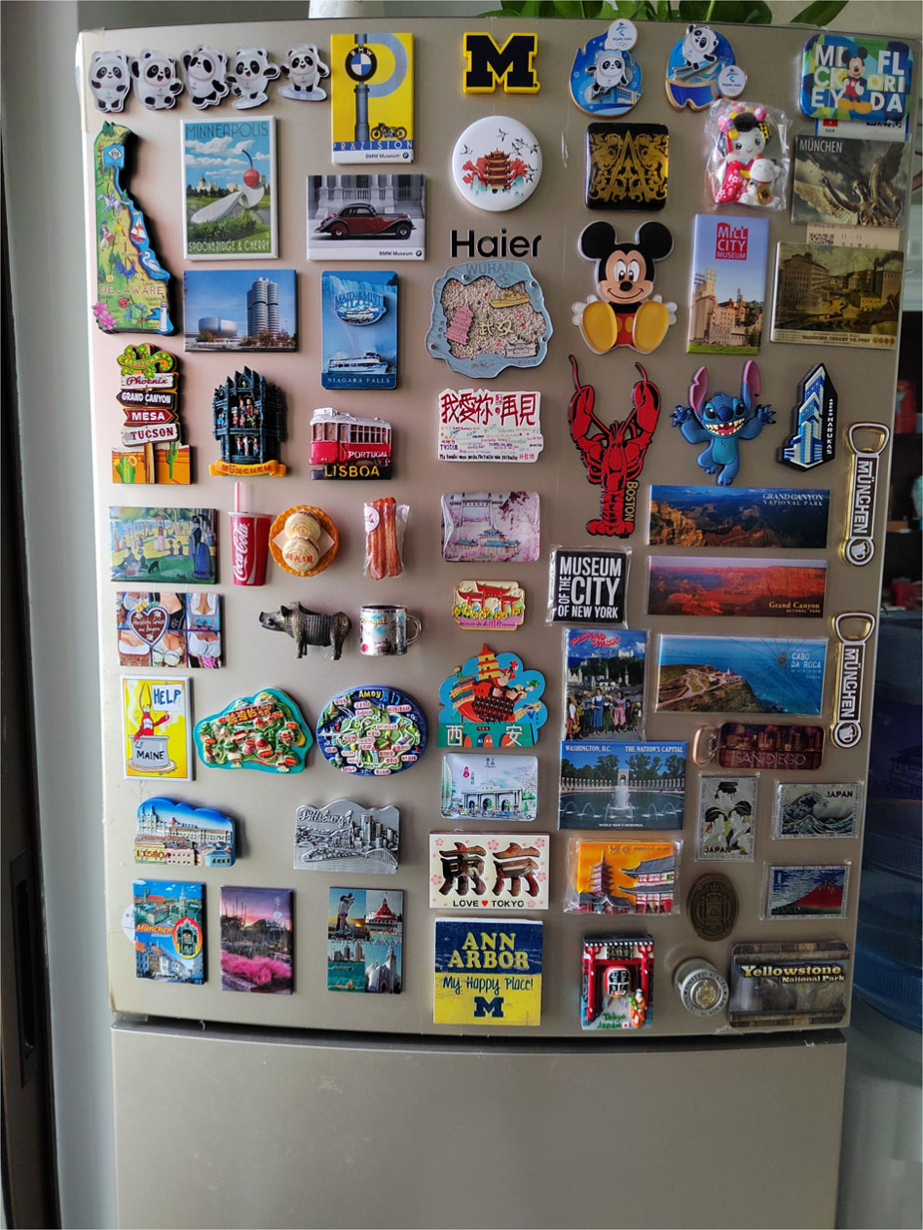
Part of the magnet collection

**16. You have published many high-quality scientific papers both domestically and internationally. In your opinion, what aspects do you think Chinese scientific journals need to strengthen in terms of high-quality development?**

I am certainly not an expert for this topic, but I will attempt to outline some of my thoughts on the matter. Firstly, I believe that Chinese scientific journals should prioritize enhancing the peer-review system and refining editorial and publishing processes. It’s important to recognize that the quality of scientific journals isn’t solely determined by the content they publish, but also by the effectiveness of their peer-review system and editorial procedures. To this end, Chinese scientific journals should strive to continually improve these areas, elevating the caliber of peer reviews, streamlining the publication timeline, and maintaining the scholarly standards and excellence of their publications.

Secondly, an increased focus on internationalization is crucial. Effective global communication and collaboration are paramount for the advancement of scientific journals. Chinese scientific journals should seek to foster collaborations with internationally acclaimed counterparts, amplify their global influence, and raise their international profile. This could be achieved through initiatives such as enhancing international collaboration projects, organizing international conferences, and attracting distinguished scholars from overseas.

Thirdly, Chinese scientific journals need to actively promote interdisciplinary research, which has become a prominent trend in contemporary scientific innovation. Encouraging cross-disciplinary exchange and integration, promoting cooperation across disciplines, and catalyzing technological advancement and growth should be a primary goal. This could be facilitated by organizing interdisciplinary symposia, inviting experts from diverse fields to contribute review articles, and similar initiatives.

Lastly, Chinese scientific journals need to enhance talent cultivation. The development of scientific journals relies on the support and management of capable individuals. Chinese scientific journals need to strengthen talent cultivation and management, enhance the qualities and capabilities of editors and peer reviewers, establish an effective incentive mechanism, and attract more top-tier professionals to participate in journal-related activities. This can be achieved through initiatives such as providing talent training, providing enticing employment packages, and establishing well-defined career development pathways, etc.

**Figure:**
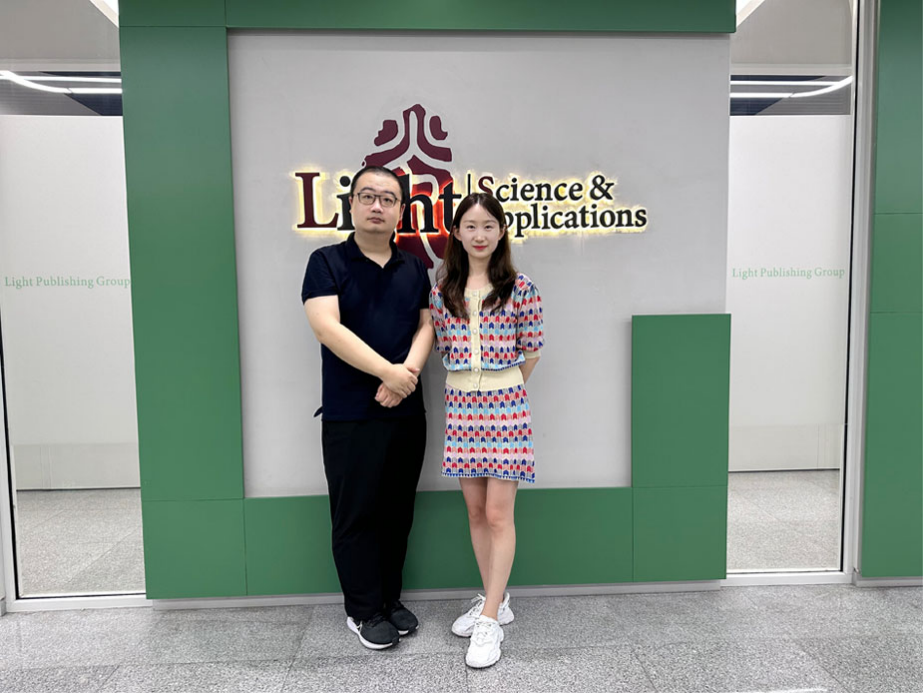
Photo at the LSA office with Dr. Siqiu Guo
